# The concept of a new two-stage fuse for high power pulse forming

**DOI:** 10.1038/s41598-022-23145-5

**Published:** 2022-10-28

**Authors:** Mikołaj Nowak

**Affiliations:** Faculty of Electrical and Control Engineering, Gdansk University of Technology, 80-233 Gdańsk, Poland

**Keywords:** Electrical and electronic engineering, Applied physics, Plasma physics, Phase transitions and critical phenomena

## Abstract

This manuscript introduces the concept, physical operating principle and studies on a new and unique two stage forming fuse (TSFF) with interstage spark gap commutation and presents its application for forming high power pulses of extreme parameters. The paper classifies TSFF performance and compares it with conventional single-stage forming fuses. The conclusions are supported by analytical and experimental studies in laboratory conditions. The design of the TSFF prototype as well as the applied measurement methods and test stands are also presented. The developed technology of the TSFF enables the achievement of unprecedented parameters of high-power pulses with overvoltages reaching 800 kV and pulse power of tens of GW in a very compact design. The unique properties of the TSFF enable its efficient integration with a wide range of energy sources, even with very limited current rising steepness or limited output voltage, which has not been possible so far with conventional single-stage forming fuses. The proposed system can be easily scaled, while ensuring much greater flexibility of applications.

## Introduction

**Figure 1:**
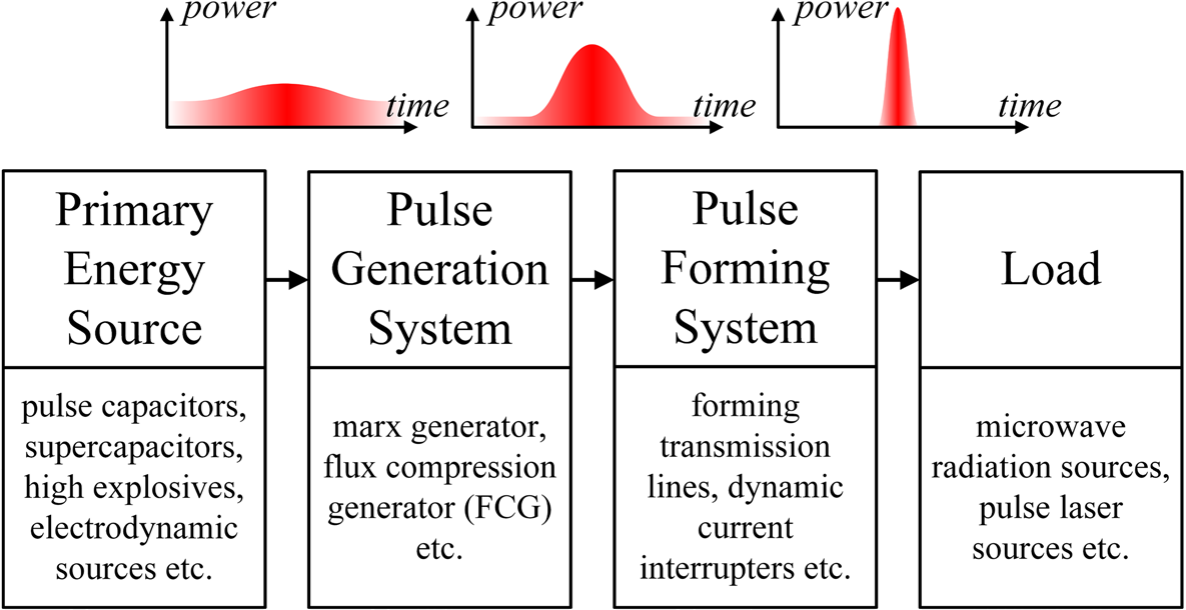
Block diagram of the high-power pulses cascade generation and forming system.

In many fields of science and engineering, there is a need to generate high power electric pulses with a significant amplitude of current (in the order of hundreds of kA) or voltage (in the order of hundreds of kV) and duration of fractions of microsecond^1^. Such pulses are used e.g. to emulate physical processes with extreme parameters in laboratory conditions (voltage or current atmospheric surge generators^2,3^, research systems for plasma or nuclear physics, e.g. X-pinch plasma generators) or as pulses supplying high power electromagnetic radiation sources, usually for radar systems^4^, pulsed laser sources^5^ or directed energy systems^6^ (counter-drone systems^7,8^, military systems^9^ etc.). High power pulse sources applications often require a compact form^10^ for transport purposes^11^, or to enable installation in a small housing (e.g. in the missile body). Direct generation of pulses of such extreme parameters with the use of a single generating stage is impossible in practice, due to significant technical difficulties (resulting from high-voltage electric or electrodynamic and thermal stresses). At the same time, individual pulse sources do not provide appropriate pulse parameters (in terms of insufficient amplitude or too long pulse duration). Therefore, in real systems, the generation of high power pulses is performed indirectly using cascade systems (as shown in Fig. 1) in which each successive stage causes a relative increase in the peak power of the pulse while reducing its duration^12^.

**Figure 2:**
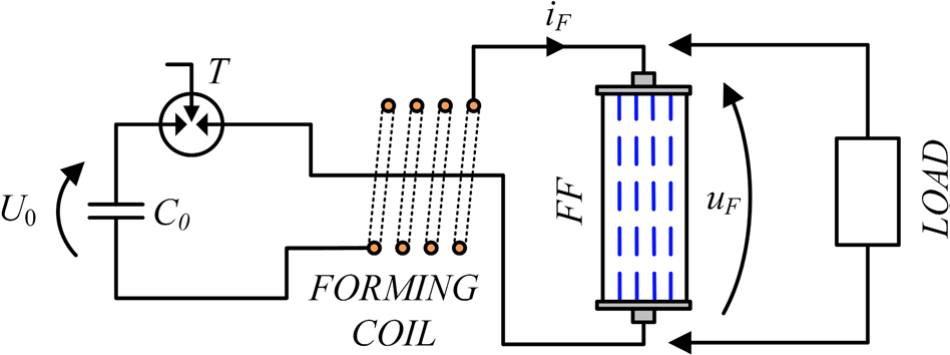
Schematic diagram of the fuse-based high-power pulse forming system supplied by pulse capacitor bank: *C*_0_—pulse capacitor bank charged to voltage *U*_0_; *T*—trigatron (triggered spark gap), *i*_*F*_, *u*_*F*_—forming fuse current and voltage.

The solutions of high power pulse generation and forming circuits can be divided into current and voltage type systems, depending on the nature of the generated pulse. Typical solutions of voltage systems are Marx generators^13–16^ or other types of voltage multiplier systems, often integrated with special forming lines^17,18^, e.g. in the Blumlein topology^19,20^. In the case of current-type generators, the most commonly used solution is the magnetic flux compression generator (FCG)^21,22^, which multiplies the current value by explosive compression of the magnetic flux coupled with the generator winding^23–25^. The FCG current output pulse is shaped in a pulse forming system (PFS) in order to adapt its parameters to load requirements. Figure 2 shows the diagram of the operating concept of a fuse-based PFS supplied from the capacitor bank. The forming process is based on the phenomenon of dynamic limitation of the current flowing in the forming inductive coil by the extremely fast opening switch which generates significant overvoltages transmitted to the load of the system. The most frequently used switching element is a forming fuse (FF)^26–28^, the operating principle of which is based on rapid disintegration of fusible elements (most often made in the form of a bundle of well-conducting wires or foil strips) due to the flow of high density currents^29^. A detailed classification, introduction to technology, and list of exemplary parameters of forming fuses for the purpose of generating high power pulses (taking into account various primary sources and pulse generation systems) have been presented in Ref.^30^.

Typical loads, also constituting the electromagnetic emission systems, cooperating with FF-based PFSs are various types of high-power microwave pulsed lamps^10^, most often: vircator, reflex triode, reditron, or hybrid systems, which due to various electronic effects (e.g., vibration of electron plasma generated by explosive emission from a cathode exposed to an extreme electric field) generate a beam of electromagnetic radiation, usually in the microwave range. For the effective generation of quasi-stable forms of electron plasma it is necessary to supply them from pulse sources (mainly wide bandwidth-type) with a significant voltage amplitude, high steepness and high output current capability^1,22^. Hence, the goal of PFS is usually to maximize the above-mentioned parameters in order to cooperate effectively with the load. Single-stage FF for applications in pulse forming systems described in the available literature present appropriate high voltage pulse generation properties, reaching values from several dozen^27,31^ to even 400 kV^32,33^, with simultaneous current limitation steepness in the order of several dozen to a little over one hundred kA/μs. The achieved instantaneous peak power values of the generated pulses reach hundreds of MW or several GW. In order to increase the power, compactness and efficiency of high power pulse generation and forming systems, it is necessary to develop a new FF solution that would go beyond the limited parameters achieved so far.

In the available literature, the so far considered criterion of the efficiency of overvoltage pulse generation in a fuse-based PFS, related to the type of fusible elements disintegration mechanism, was the maximum value of the current density *j*_*max*_ in the fusible elements cross-section^34,35^. Extensive research on the fuse-based PFS (the schematic diagrams as in Fig. 2) has led to the unequivocal identification of an extended criterion determining the efficiency of high-power pulse generation in the PFS as the maximum steepness of the current density *dj/dt*_*max*_ in fusible elements cross-sections. The performed and systematized analysis of the phenomena occurring in the FF in a wide range of operating conditions has led to the question of the possibility of increasing the value of the aforementioned criterion (*dj/dt*_*max*_) in fuse-based systems above the values achieved so far, which could provide an opportunity for significant increase in the steepness of current limitation, and thus for the formation of overvoltages with significantly greater amplitude than in the case of conventional FF solutions. Increasing the steepness of the fuse current density *dj/dt* in the circuit with the forming induction coil (Fig. 2) is possible by reducing the fusible elements equivalent cross-section (related to the number of parallel elements and the cross-section of a single fusible wire) or by increasing the voltage of the source forcing the current flow in the circuit. However, direct application of the former method simultaneously limits the steepness of the current rising slope in the LC circuit by inserting a significant fuse equivalent resistance and limiting the current value preceding the fuse disintegration—the so-called pre-arcing current *i*_*p*_ (due to a smaller value of the Joule integral of the fuse operation^36^), the value of which determines the steepness of the current limitation to zero by FF. On the other hand, increasing the voltage of the source supplying the current flow in the PFS is related with the need to use capacitor banks with a higher operating voltage, which in practice causes insulation problems in the construction of the test stand, higher complexity of the battery pre-charging systems, and significant reduction in the compactness and mobility of this type of solution.

This article proposes a concept of a new and original solution of TSFF with inter-stage spark gap commutation which allows to increase the power and decrease the duration time of the formed pulses, and thus to increase the steepness of the current density rise in the PFS active element, i.e. the FF, with simultaneous possibility of integrating this type of system with primary energy sources (e.g. capacitor banks) with limited output voltage, or with current-type sources (e.g. FCG).

With the use of a TSFF, it is possible to increase the efficiency of the high-power pulse forming process by multiple times compared to conventional systems based on single-stage FFs and, therefore, to increase the amplitude of the generated voltage pulse to the value reaching 800 kV with current limitation steepness above 300 kA/μs, and simultaneously to limit its duration time to tenths or hundredths of μs. Systems with such parameters which would maintain high power density, energy density, and compactness of the solution, have not been documented so far.

## The concept and principle of operation of the two-stage forming fuse

**Figure 3:**
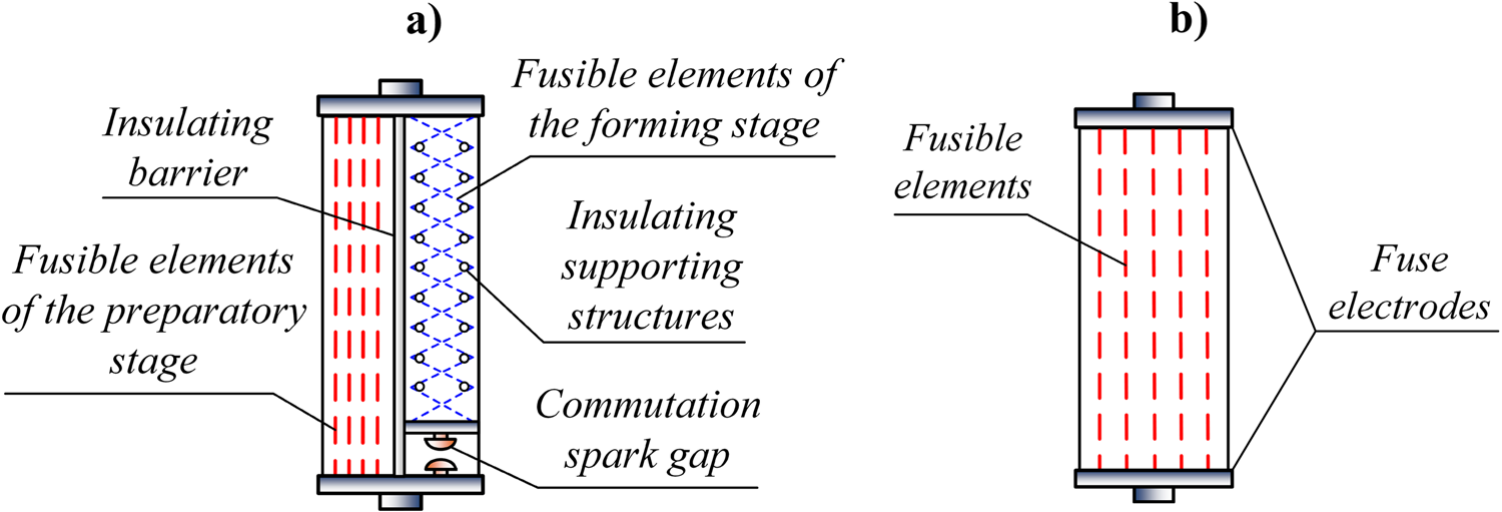
Comparison of the TSFF (**a**) and the single-stage FF (**b**) structure.

The principle of operation of the TSFF (as shown in Fig. 3a) is based on increasing the steepness of the current density rise in stage II, hereinafter referred to as the *forming stage*, with a relatively small equivalent cross-section of the fusible elements, due to the commutation of significant current from stage I of the TSFF, called the *preparatory stage*, with larger cross-section of fusible elements. Due to the significant increase in steepness of the current density rise in the forming stage fusible elements (even by three orders of magnitude in relation to the preparatory stage) after commutation, a homogeneous electro-explosion of the forming stage fusible elements occurs and the PFS current is extremely rapidly limited, which generates a significant overvoltage in the circuit with the forming induction coil.

The structure of the TSFF is presented in a simplified way in Fig. 3a and compared to a conventional single-stage forming fuse shown in Fig. 3b. The preparatory stage fusible elements are directly connected to the TSFF electrodes. The forming stage is connected in series with the commutating spark gap (CSG) and the entire branch is connected in parallel to the preparatory stage. The fusible elements of both stages are placed in one fuse compartment and separated by an insulating barrier preventing ignition of an arc between them.

The concept of the TSFF is directly related to the operating principle of the pulse forming systems, which consists in increasing the peak power and reducing the pulse duration by each successive stage of the system. The purpose of the fuse preparatory stage is to allow the current to flow in the PFS forming inductance in the initial phase of operation (the stage of current rising). For this reason, it consists of a great number of parallel fusible elements with a large equivalent cross-section *n*_1_*S*_1_, the value of which results from the specific Joule’s integral determining the maximum value of the current waveform. The forming stage fusible elements, with a smaller equivalent cross-section *n*_2_*S*_2_, are separated by CSG from the primary current flow path in the initial phase of operation. The process of disintegration of preparatory fusible elements initiates the current limitation phenomenon and the appearance of overvoltage between CSG electrodes, as a result of which the discharge is ignited and the current is rapidly commutated to the forming stage. Rapid commutation of high-value current to the forming stage generates very high density and steeply rising current flows in its fusible elements, which leads to their electro-explosion. At the same time, during the preparatory stage zero-current interval after inter-stage current commutation (in fact, a very small current may flow through the preparatory stage at this time), the thermal ionization process in the plasma channel, formed after disintegration of the preparatory stage fusible elements, stops and its partial deionization takes place. As a result, the preparatory stage plasma channel is able to rebuild the electrical strength before the moment of sudden current limitation to zero by the forming stage fusible elements and the generation of a significant overvoltage pulse, which (in conventional single-stage FF) could cause the fuse post-disintegration discharge reignition and make the current limitation process ineffective.

The equivalent cross-section of the preparatory stage fusible elements should be selected so that their disintegration takes place just before the current waveform reaches its maximum. Hence, the selection of the number, cross-section and length of the fusible elements is usually based on the energy criterion and the Joule integral criterion, as presented in Ref.^30^. However, due to the strong non-linearity of the phenomena occurring in the TSFF and its influence on the PFS circuit, this method is approximate and to effectively determine the optimal operating conditions it is necessary to use an experimental method. Since there is no need to generate very high overvoltages by the preparatory stage (which should only ensure the ignition of CSG), it is possible to supply the TSFF-based PFS from sources which are not able to ensure high dynamics of current density rise, without significant impact on the value of the overvoltage formed by the forming stage. In fact, it is the preparatory stage which in any case ensures the appropriate, high dynamics of current density rise for the forming stage fusible elements.

**Figure 4:**
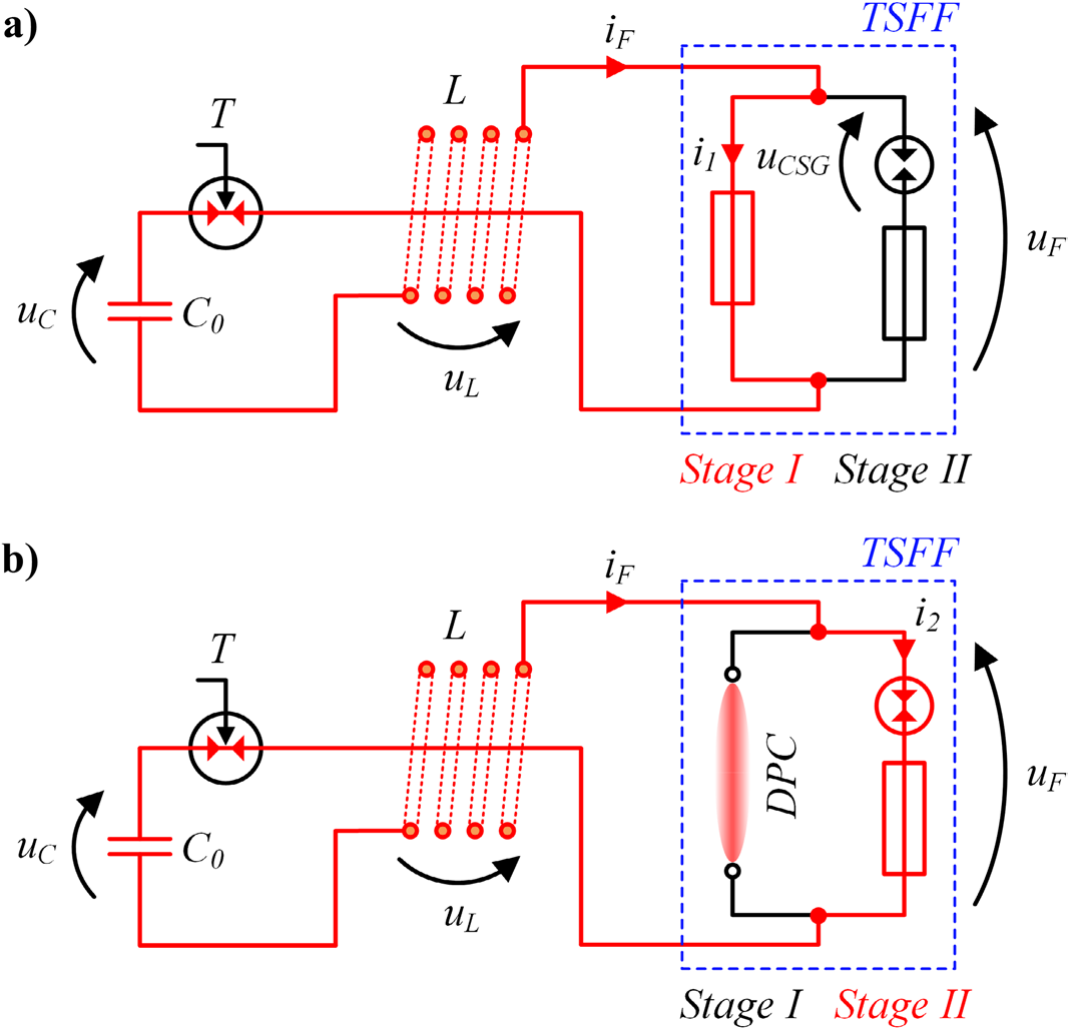
Schematic diagram of the TSFF-based PFS during operation in the preparatory phase (**a**) and in the pulse forming phase after inter-stage commutation (**b**): *C*_0_—pulse capacitor bank with voltage *u*_*c*_, *T*—trigatron (triggered spark gap), *L*—forming inductance, *DPC*—deionizing plasma channel, *i*_*F*_, *u*_*F*_—fuse current and voltage, *i*_1_, *i*_2_—preparatory and forming stage current.

**Figure 5:**
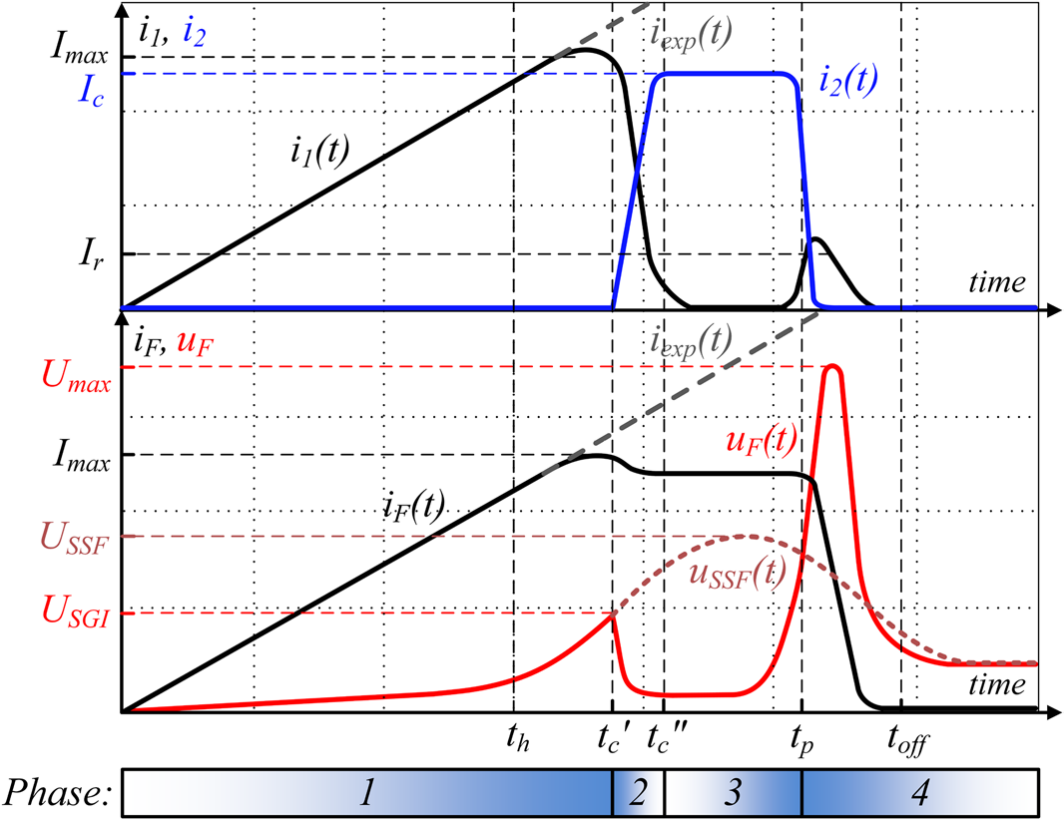
Idealized waveforms of electrical quantities during TSFF operation, with marked characteristic values (symbols in accordance with Fig. 4 and the descriptive text). Illustrative drawing not to scale.

Figure 4 presents a simplified schematic diagram of the PFS with a two-stage forming fuse in two operating stages. Typical, idealized waveforms of electrical quantities, i.e. TSFF currents and voltages in PFS, are shown in Fig. 5.

The TSFF operation process can be divided into four phases:

I. Current rising through the preparatory stage until its disintegration, and generating an initial overvoltage for CSG discharge ignition.
II. Current commutation from the preparatory stage to the forming stage after disintegration of preparatory stage fusible elements.
III. Current flow through the forming stage with simultaneous electric strength recovery of a plasma channel formed after the disintegration of preparatory stage fusible elements (zero-current interval).
IV. Rapid limitation of the current by electro-explosion of forming stage fusible elements. Generation of significant overvoltage in the forming coil.

**Figure 6:**
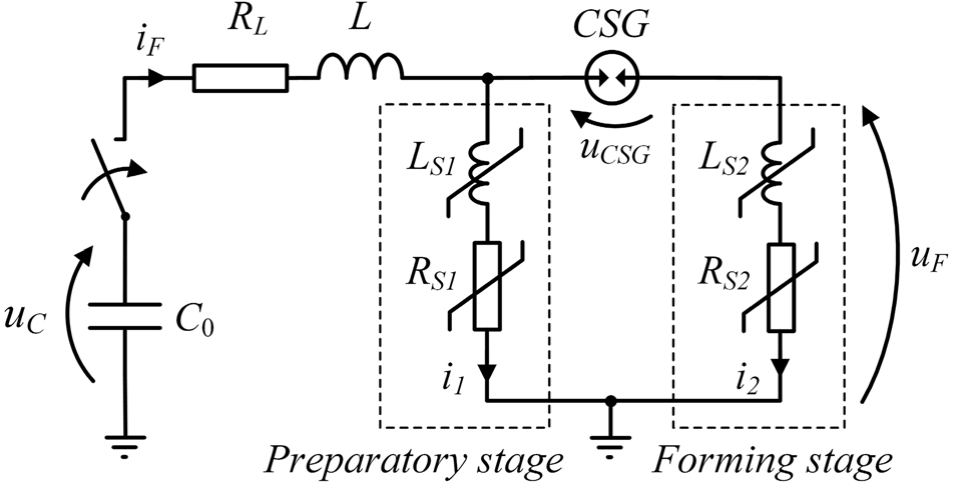
**S**implified TSFF-based PFS lumped circuit model (refer to the descriptive text for explanation of symbols).

Figure 6 presents a simplified TSFF-based PFS circuit model, including lumped peripheral elements representing both stages of the fuse, on the basis of which it is possible to analyze the system operation.

Like in the single-stage FF design, phase 1 of TSFF operation begins at the moment of current flow initiation from the source (capacitor bank or FCG) in the forming circuit, as shown in Fig. 6. In the initial phase, the total fuse current *i*_*F*_ flows entirely through the elements of the preparatory stage *i*_1_, causing an increase in their internal energy, temperature and, as a result, resistance. Until the preparatory stage fusible elements reach the level of internal energy necessary to initiate the process of their melting (at time *t*_*h*_) and disintegration (which limits the current in comparison to the expected discharge current of the capacitor bank *i*_*exp*_), TSFF has the features of a single-stage FF due to galvanic separation of the forming stage by CSG. At the initiation moment of the process of disintegration of preparatory stage fusible elements, their equivalent resistance increases rapidly and, as a result, the voltage drop in the TSFF preparatory stage increases until the CSG ignition voltage *U*_*SGI*_ is reached. Due to a significant increase in the resistance of the preparatory stage elements, the voltage drop across the CSG is mainly resistive. Thus, in phase 1 of TSFF operation, the voltage drop on the CSG results directly from the state of the preparatory stage fusible elements and is determined by the relation (1).

1 $${u}_{CSG}\left(t\right)={R}_{S1}{i}_{F}+{L}_{S1}\frac{d{i}_{F}}{dt}\approx {R}_{S1}{i}_{F},$$

where *R*_*S1*_, *L*_*S1*_ resistance and inductance of the preparatory stage.

As soon as the voltage drop on the CSG reaches the structurally determined value of the ignition voltage of the spark gap at time *t*_*c*_*'*, the commutation of the current to the forming stage begins. As a result of the initiated phenomenon of disintegration of preparatory stage fusible elements, the current flowing in the circuit is limited to some extent. The limitation factor *γ*_*i*_, defined as the ratio of the current commutated to the forming stage *I*_*c*_ referred to the maximum value of the preparatory stage current *I*_*max*_ (2), results from the ignition voltage *U*_*SGI*_ at which the current is commutated and is related to the state of the preparatory stage fusible elements at the time when the CSG is ignited.

2 $$ \gamma _{i}  = f\left( {U_{{SGI}} } \right) = \frac{{I_{c} }}{{I_{{\max }} }}, $$

where *I*_*max*_ maximum value of the TSFF preparatory stage current.

At time *t*_*c*_*'* of CSG ignition, the commutation of the current from the preparatory stage to the forming stage begins due to the much lower value of the equivalent resistance of the “cold” fusible elements of the forming stage. In the beginning of the commutation process, the forming stage fusible elements have an ambient temperature, unlike the (high-resistance) plasma column formed after the disintegration of the preparatory stage fusible elements.

The dynamics of the interstage current commutation process in the time interval *t*_*c*_*'* to *t*_*c*_*''* can be described by Eq. (3) with approximate initial conditions (4). The presented peripheral model, for the purpose of qualitative analysis of the processes taking place during the operation of TSFF, has been simplified and limited to the main elements, without taking into account the minor parasitic elements of the TSFF. In practice, the time constants (of the order of ps) resulting from the existence of e.g. distributed capacities of TSFF structures and current paths are unnoticeable from the perspective of the relatively “long” ns-scale time constants of thermoelectric and thermo-magneto-hydrodynamic processes.

It can be assumed that during the commutation there is no significant increase in the resistance of the forming stage elements, thus *R*_*S2*_ ≈ *const.* while the increase of resistance *R*_*S1*_ continues, hence the inductance value of the fuse preparatory stage *L*_*S1*_ is negligible. In such a short time, the voltage across the source capacitor *u*_*C*_ remains constant. After arc ignition in CSG, the voltage drop *u*_*CSG*_ is also negligible and does not affect the commutation process.

3 $$\frac{L\cdot {L}_{S2}}{{R}_{S1}\left(t\right)\cdot {R}_{S2}}\frac{{d}^{2}{i}_{2}}{d{t}^{2}}+\left(\frac{{R}_{L}{L}_{S2}}{{R}_{S1}\left(t\right){R}_{S2}}+\frac{L}{{R}_{S1}\left(t\right)}+\frac{L}{{R}_{S2}}+\frac{{L}_{S2}}{{R}_{S2}}\right)\frac{d{i}_{2}}{dt}+\left(\frac{{R}_{L}}{{R}_{S1}\left(t\right)}+\frac{{R}_{L}}{{R}_{S2}}+1\right){i}_{2}=\frac{{u}_{C}}{{R}_{S2}},$$

4 $$ i_{2} \left( {t = t_{c}^{\prime } } \right) = 0, \frac{{di_{2} }}{dt}\left( {t = t_{c}^{\prime } } \right) \approx \frac{{U_{SGI} }}{{L_{S2} }}, $$

where *L* forming inductance, *R*_*S*2_*, L*_*S*2_ forming stage resistance and inductance.

Apart from the parameters of the commutation circuit, the key parameters determining the dynamics of interstage commutation, and resulting from the initial conditions of the commutation process, are the ignition voltage *U*_*SGI*_ of the commutation spark gap and the fuse forming stage inductance. Taking into account this fact and that the current density in the fusible elements decreases with the increasing number of parallel elements, it can be concluded that there is a certain optimum number of fusible elements of the forming stage in relation to the preparatory stage. Optimization studies are underway to determine the optimal operating parameters of the TSFF. Due to the significant non-linearity of the Eq. (3) parameters, the solution can be determined numerically using, for example, the resistive model of the forming fuse, as in Ref.^30^ or Ref.^12^.

The process of current commutation from the preparatory to the forming stage ends at time *t*_*c*_*''* at which the entire fuse current flows through the forming stage, *i*_*F*_ = *i*_2_. From now on (phase 3 of TSFF operation), for a short time required to increase the internal energy of the forming stage fusible elements, the voltage *u*_*F*_ on the fuse reaches a small value resulting from the voltage drop on their equivalent initial resistance (resistance of the elements at a near-to-ambient temperature). Depending on the number of fusible elements of the forming stage and the value of the voltage *u*_*C*_ remaining on the capacitance *C*_0_, it is possible to notice temporary increase, settle, or decrease of the fuse current value, according to Eq. (5).

5 $$\frac{d{i}_{2}}{dt}=\frac{1}{{L}_{S2}}\left({u}_{C}-{R}_{S2}{i}_{2}\right).$$

Regardless the commutation of the current from the preparatory to the forming stage, the process of disintegration of the preparatory stage fusible elements does not stop. After the current is commutated to the forming stage, further radial expansion of the plasma channel in the preparatory stage (which results in an increase in the diameter of the plasma column) and its intensive deionization in no-current conditions (zero-current interval) take place, thus further increasing the equivalent resistance *R*_*S1*_.

Once the forming stage fusible elements have reached the necessary internal energy at pre-arcing time *t*_*p*_, the current is rapidly limited to zero by electro-explosion of the forming stage fusible elements, and the overvoltage of extreme value *U*_*max*_ occurs on the fuse. Such a high overvoltage is possible due to the following features of the forming stage elements:

- A much higher value and steepness of the current density increase in the forming stage fusible elements (due to their smaller equivalent cross-section) leads to their higher disintegration dynamics, i.e., ensures faster radial expansion of the plasma column, as a result of which the equivalent channel resistance also increases faster.
- The fusible elements of the forming stage may have a much greater length than those of the preparatory stage, so that they can disintegrate with the separation of a greater number of elementary arc gaps, i.e., at a higher voltage value of the multi-arc column.
- Due to the smaller equivalent cross-section of the forming stage fusible elements, the plasma channel created as a result of their rapid electro-explosion has a lower density of free charge carriers, therefore its equivalent resistance is higher and its deionization process occurs with greater dynamics.

During the final current limiting process, the voltage of the forming stage appears also on the plasma column of the preparatory stage, which during the zero-current interval *t*_*p*_* − t*_*c*_*''* was able to freely restore the electric recovery strength. Due to the extreme levels of generated overvoltages and much faster equivalent resistance increase in the fuse forming stage, the return leakage current begins to flow through the plasma column of the preparatory stage, reaching the maximum value *I*_*r*_, and, by partially conducting the current limited by the forming stage fusible elements, can cause certain reduction of the generated overvoltage. Due to the deep deionization state of its plasma channel, along with appropriate selection of fuse parameters, the return current of the preparatory stage does not lead to its re-ionization, which results in effective current limitation to zero at time *t*_*off*_.

However, if the zero-current interval of the preparatory stage is provided too short, it is possible to re-ionize still deionizing plasma channel, as a result of which the high-value follow current (discharging the energy remaining in the capacitor bank) can flow. In that case, the current limitation and pulse forming process may be ineffective.

Therefore, it is necessary to minimize the value of the return current *I*_*r*_ by appropriate selection of the cross-sections *n*_1_*S*_1_ of the preparatory stage and *n*_2_*S*_2_ of the he forming stage, along with the value of the CSG ignition voltage *U*_*SGI*_, ensuring the appropriate length of the forming stage elements and providing sufficiently long deionization time *t*_*p*_ − *t*_*c*_*''* of the preparatory stage plasma channel. The entire current limiting process in the interval *t*_*off*_ − *t*_*p*_ should be completed as soon as possible to maximize the efficiency of the pulse forming process.

## Two-stage forming fuse parameters

A two-stage forming fuse, in terms of principle and effectiveness, has properties that are impossible to achieve in a single-stage FF of any design. In the case of a single-stage FF, during the initial phase of operation, the fusible elements should guarantee the possibility for the forming inductance current to rise to a specific, often large value (in the order of hundreds of kA and even MA^24^). Therefore, they should have a very low conduction resistance and provide the highest possible value of the pre-arcing Joule integral *I*^*2*^*t*_*p*_, which is the measure of the energy density applied to the fusible elements, determined by the type of fusible material and the square of the product of the cross-section *S* and the number of fusible elements *n*, according to (6).

6 $${I}^{2}{t}_{p}=\underset{0}{\overset{{t}_{p}}{\int }}{i\left({t}^{^{\prime}}\right)}^{2}d{t}^{^{\prime}}\approx {K}_{M}{\left(n\cdot S\right)}^{2},$$

where: *K*_*M*_ Meyer constant or specific integral of the fusible material (for copper: *K*_*MCu*_ ≈ (1.2 ÷ 1.4)‧10^17^ A^2^s/m^4^, or silver: *K*_*MAg*_ ≈ (0.8 ÷ 1)‧10^17^ A^2^s/m^4^)^37^.

It is worth mentioning that the Meyer constant *K*_*M*_ under the conditions of rapid heating of fusible elements in FF associated with a significant steepness of the fuse current density growth *dj/dt* may reach slightly higher values^29,38^ than the design values adopted in the literature.

The fuse equivalent resistance is proportional to the length of the fusible elements and inversely proportional to their equivalent cross-section. In order to obtain a sufficiently high PFS current, it is necessary to minimize the fuse resistance in the conduction state by minimizing the length and maximizing the cross-section and number of fusible elements.

On the other hand, after reaching the maximum value by the PFS current, it should be rapidly reduced to zero, so the fuse resistance should reach the highest possible value in the shortest possible time (which is determined by the fuse disintegration mechanism^29,39^). One of the key factors determining the type of the fusible elements disintegration mechanism is the maximum value of the current density *j*_*max*_ and the steepness of the current density rise *dj/dt*. Due to the above-mentioned conditions, in the second phase of FF operation (i.e., the current limiting phase), in order to obtain the greatest possible dynamics of the current limitation to zero process and the fastest possible electric strength recovery, efforts should be made to minimize the cross-section and number of parallel fusible elements and to maximize their length.

The above criteria for the selection of fusible elements, deciding on the effectiveness of pulse forming by the FF, are opposite. Therefore, the selection of an appropriate number, cross-section, and length of single-stage FF elements is not able to guarantee the optimal fuse operating conditions in the PFS. At the same time, the design of TSFF enables functional adjustment of fusible elements in both stages of the fuse (preparatory and forming stage) to the operating conditions in the time of current rising and the time of switching it off. Both TSFF fuse stages have the number, cross-section, and length of fusible elements adapted to the operating phases in PFS, i.e. a significant number of preparatory stage elements with larger cross-section and limited length ensure the possibility of increasing the current in the PFS forming inductance to the highest possible value, while a small number of forming elements with smaller diameter and increased length ensures quick current limitation and, consequently, much greater efficiency of the pulses generation process. Due to the incomparably higher value of the current density and steepness of its rise in the forming stage fusible elements, compared to the preparatory stage, their disintegration is of a homogeneous explosion nature in accordance with the criterion (7)^38,40,41^.

7 $${t}_{r}<{\tau }_{I},$$

where *t*_*r*_ time to fuse disintegration, *τ*_*I*_ time constant of the development of surface and internal instabilities in the fusible element, which can be estimated using (8)^42,43^, approximately independent of the length and winding geometry of the fusible elements.

8 $${\tau }_{I}\approx \frac{4\pi \sqrt{\frac{\rho }{{\mu }_{0}}}}{{j}_{max}},$$

where *ρ* fusible material density, *μ*_0_ absolute vacuum magnetic permeability, *j*_*max*_ maximum value of the fusible element current density at time before disintegration.

**Figure 7:**
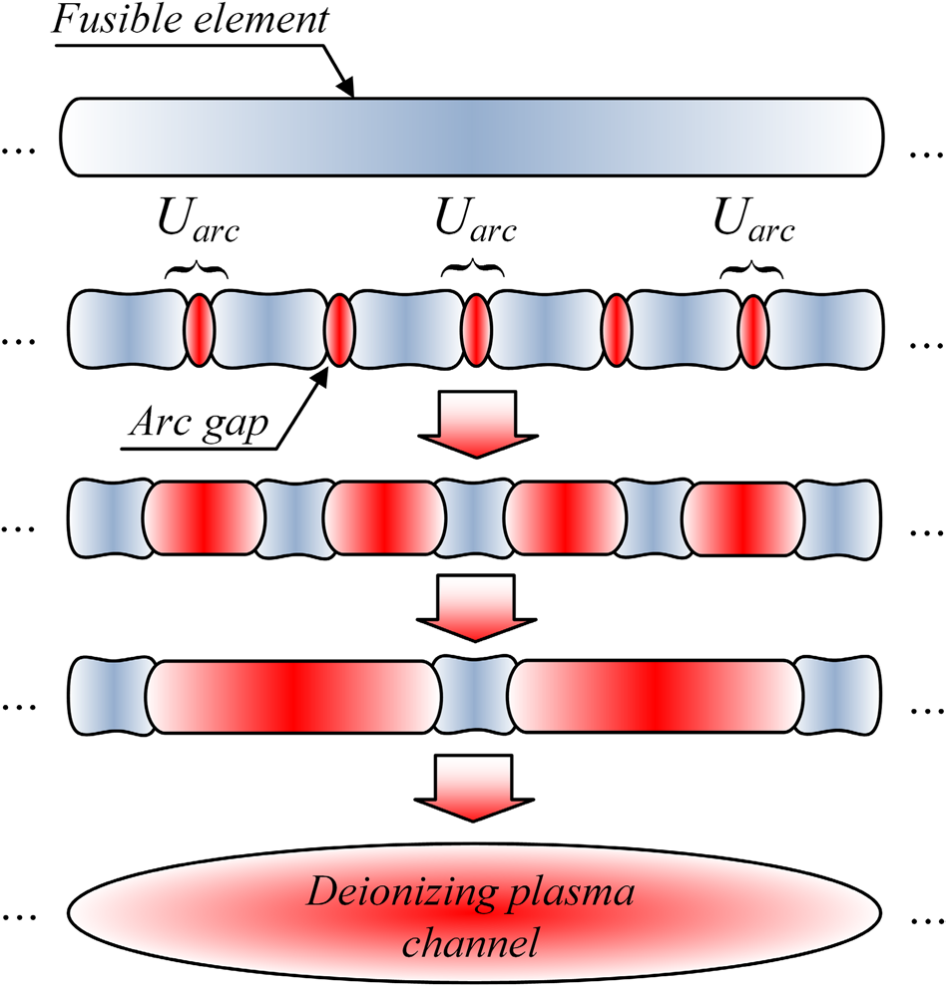
Schematic drawing of the electro-explosive striated disintegration process of the fusible element under the conditions of a significant current density steepness.

For the values of currents and times considered in this paper, the time constant *τ*_*I*_ of the development of surface and internal instabilities reaches values ranging from hundreds of ns to single μs. Therefore, to ensure homogeneous nature of the explosion of fusible elements, the time to their disintegration should be shorter than *τ*_*I*_. The process of electro-explosive disintegration of fusible elements under conditions of steep increase in current density with the isolation of elementary arc gaps (striated fuse-wire disintegration due to electrodynamic and thermodynamic forces^29,39^), accompanied with plasma channel forming and deionization, is shown in Fig. 7.

The design of the TSFF is a combination of two single-stage FF fuses with features adapted to the two phases of fuse operation. The forming stage elements have a much smaller equivalent cross-section *n*_2_*S*_2_ and a greater length *l*_2_, as compared to the preparatory stage, which is expressed by the relations (9). Increasing the length of the forming stage fusible elements, while maintaining the specified overall dimensions of the TSFF fuse chamber, may be possible by spiral winding of the elements or winding along a polygonal chain on insulating supports, as shown in Fig. 3a.

9 $${n}_{1}{S}_{1}\gg {n}_{2}{S}_{2}\, oraz \,{l}_{2}\gg {l}_{1}.$$

If it is necessary to achieve the TSFF Joule integral value equivalent to a single-stage FF, the number of parallel preparatory stage elements is reduced in relation to the single-stage FF and the resulting deficit is compensated with the value of the Joule integral of the forming stage elements.

Due to the significantly higher steepness of the current density increase *dj/dt* in the fusible elements of the forming stage, it is necessary to use a winding wire with a correspondingly smaller diameter to limit the phenomenon of magnetic field diffusion inside the conductor. Too large diameter of fusible elements in combination with a sufficiently short interstage commutation time may result in inhomogeneous surface heating and ablative explosion of the fusible element^29^, which occurs when the time necessary to evaporate the entire fusible material is much shorter than the electro-magneto-thermal time constant of the radial current density diffusion inwards the fusible element.

Due to the current diffusion phenomenon and limited (many orders of magnitude lower) thermal diffusion speed, the evaporation of the fusible material during ablative explosion initially occurs only on the conductor surface, often even before the fusible element core reaches the liquid state. The process of this type takes place in conductors of considerable diameter in relation to the skin-depth of current penetration subjected to the flow of currents of considerable density and high dynamics. Ablative explosion of a conductor is much less dynamic than homogeneous explosion, therefore in the case of FF it is an undesirable phenomenon and the risk of its occurrence should be limited by using fusible elements with a suitably small diameter.

**Figure 8:**
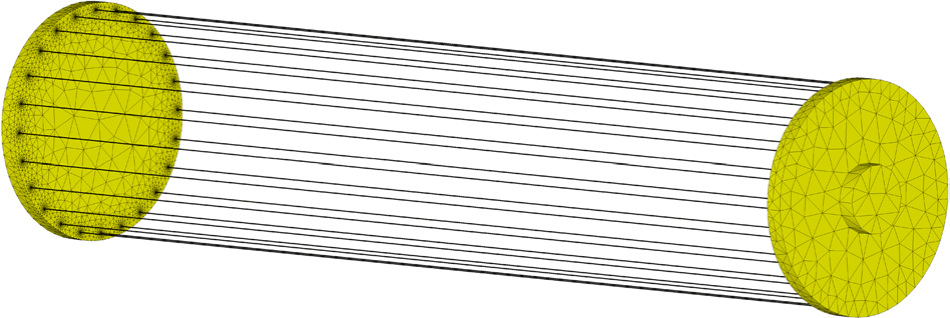
Discretized model of the interior of the fuse compartment consisting of 24 parallel fusible elements (300 mm long and 0.25 mm or 0.125 mm in diameter).

In order to visualize the effect of the fusible element diameter on the internal distribution of current density, numerical simulations of the current flow in conductive elements of the fuse compartment (Fig. 8) consisting of 24 parallel fuses with diameters of 0.25 mm and 0.125 mm have been simulated in the transient state as a response to a forcing current with significant linear steepness (exceeding 100 kA/μs in a single fuse). The simulation tests were performed in time domain using the finite element method (based on the magneto-thermal diffusion and Maxwell equations and taking into account the coupling of electromagnetic and thermal fields) in the CST Studio 2022 computing environment^44^.

**Figure 9:**
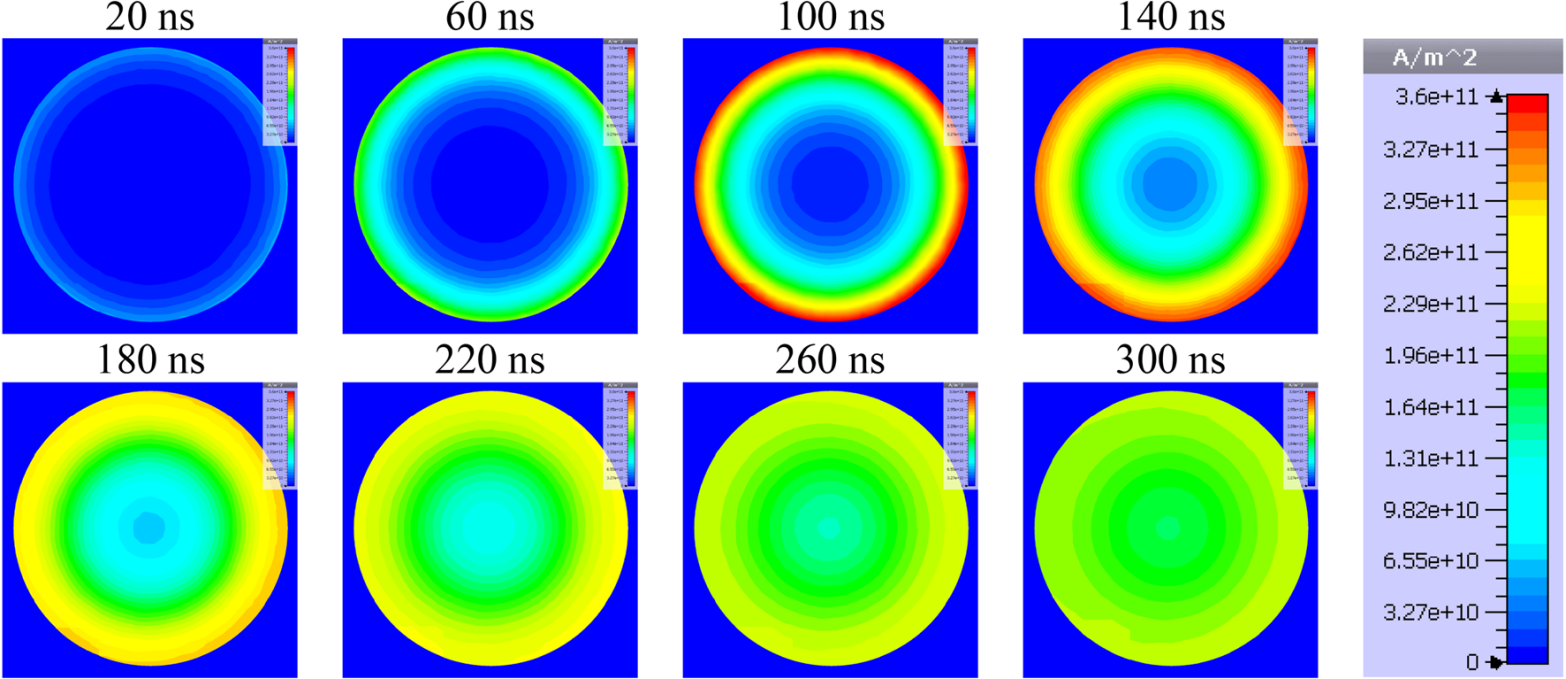
Visualization of dynamic distribution of current density in the cross-section of a silver fusible element with a diameter of 0.25 mm during the flow of current with significant steepness.

**Figure 10:**
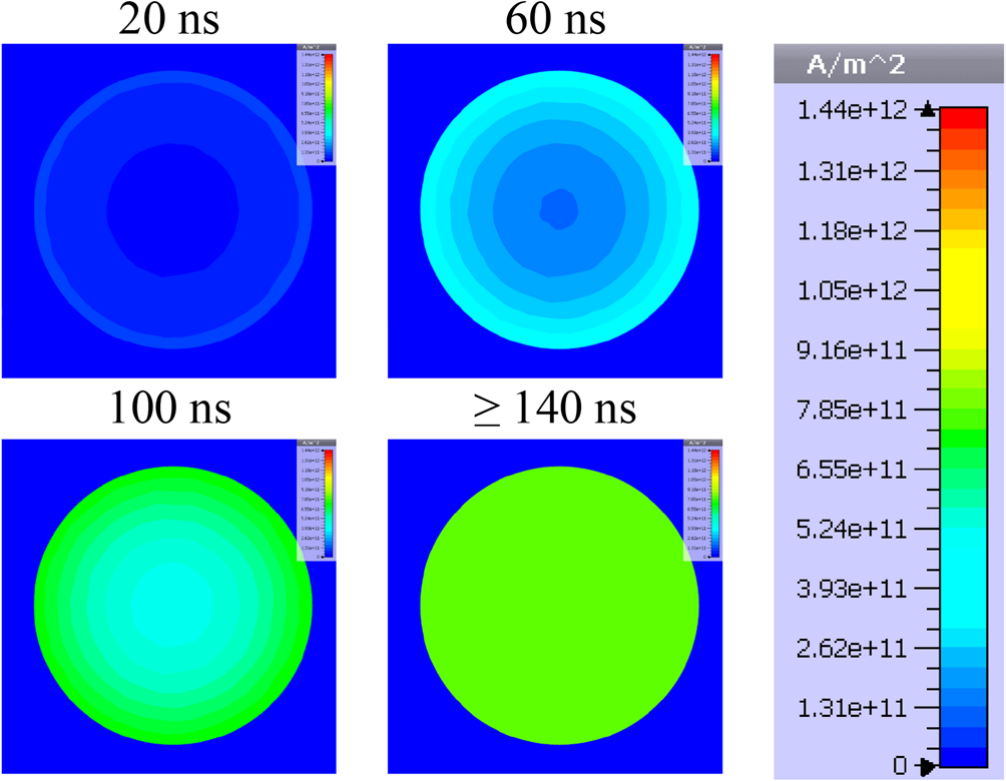
Visualization of dynamic distribution of current density in the cross-section of a silver fusible element with a diameter of 0.125 mm during the flow of current with significant steepness.

Figures 9 and 10 present visualized distributions of the absolute value of current density *j* in the cross-section of a single fusible element with a diameter of 0.25 mm and 0.125 mm, respectively, at selected times of current increase.

Figures 9 and 10 show that, according to the assumptions, the influence of the fusible element diameter on the current density distribution is a factor which should be taken into account in the case of current pulses with a significant rising steepness (as in the case of interstage commutation). Uneven current density distribution may change the nature of the fuse disintegration process and deteriorate the PFS generation properties. That is why in the TSFF forming stage, fusible elements made of a wire with smaller diameter than that in the preparatory stage should be used.

On the basis of the representative TSFF current curves (Fig. 5), it is possible to develop approximate dependencies determining the values of Joule integrals for the preparatory stage *I*^2^*t*_1_ and the forming stage *I*^2^*t*_2_ according to (10) and (11), respectively. For simplicity, a triangular waveform of current *i*_1_ and a trapezoidal waveform of *i*_2_ was assumed. Designations consistent with Fig. 5 were adopted.

10 $$ I^{2} t_{1} = K_{M} n_{1}^{2} S_{1}^{2} \approx \frac{1}{3}I_{\max }^{2} t_{c}^{^{\prime\prime}} , $$

11 $${{I}^{2}t}_{2}={K}_{M}{n}_{2}^{2}{S}_{2}^{2}\approx {\gamma }_{i}^{2}{I}_{\mathrm{max}}^{2}{t}_{2AV},$$

where *γ*_*i*_ current limitation factor, defined as (2), *t*_*2AV*_ average time of the current flow through the forming stage elements defined as (12).

12 $$ t_{2AV} \approx \frac{1}{2}\left( {2t_{off} - t_{c}^{^{\prime}} - t_{c}^{^{\prime\prime}} } \right), $$

where *t*_*off*_ current limitation to zero time. Designations in accordance with Fig. 5 were adopted.

Based on Fig. 5, it is also possible to determine the approximate average values of the steepness of current density rise in the fusible elements of the preparatory stage *dj*_*1*_*/dt*_*AV*_ and the forming stage *dj*_2_*/dt*_*AV*_, Eqs. (13) and (14), respectively.

13 $$ \frac{{dj_{1} }}{dt}_{AV} = \frac{{I_{1AV} }}{{n_{1} S_{1} \cdot t_{1AV} }} \approx \frac{{I_{max} }}{{2n_{1} S_{1} \cdot t_{c}^{^{\prime\prime}} }}, $$

14 $${\frac{d{j}_{2}}{dt}}_{AV}=\frac{{I}_{cAV}}{{n}_{2}{S}_{2}\cdot {t}_{2AV}}\approx \frac{{\gamma }_{i}{I}_{\mathrm{max}}}{{n}_{2}{S}_{2}\cdot {t}_{2AV}}.$$

The ratio of the steepness of the forming stage and preparatory stage current density dynamics can be determined from Eq. (15).

15 $$ \frac{{\frac{{dj_{2} }}{dt}_{AV} }}{{\frac{{dj_{1} }}{dt}_{AV} }} \approx \frac{{2\gamma_{i} \cdot n_{1} S_{1} \cdot t_{c}^{^{\prime\prime}} }}{{n_{2} S_{2} \cdot t_{2AV} }} . $$

On the basis of Eqs. (10) and (11), it is possible to determine the times of current flow through the fusible elements *t*_*c*_*''* and *t*_*2AV*_, and simplify Eq. (15) to a form depending only on the ratio of the equivalent cross-sections of the preparatory and forming stage elements (16). It should be noted that the Meyer constant *K*_*M*_ (6) may have different values for different steepness of current density *dj/dt*, but in the range of variability it is limited upwardly by twice its initial value^29,38^.

16 $$\frac{{\frac{d{j}_{2}}{dt}}_{AV}}{{\frac{d{j}_{1}}{dt}}_{AV}}\approx \frac{2{\gamma }_{i}\cdot {n}_{1}{S}_{1}\cdot \frac{3{K}_{M}{n}_{1}^{2}{S}_{1}^{2}}{{I}_{max}^{2}}}{{n}_{2}{S}_{2}\cdot \frac{{K}_{M}{n}_{2}^{2}{S}_{2}^{2}}{{\gamma }_{i}^{2}{I}_{max}^{2}}} =6{\gamma }_{i}^{3}\cdot {\left(\frac{{n}_{1}{S}_{1}}{{n}_{2}{S}_{2}}\right)}^{3}.$$

The actual value of the current limitation factor *γ*_*i*_ is in the range from 0.5 to 1 and depends mainly on the CSG ignition voltage *U*_*SGI*_. It can be concluded that in order to disintegrate the forming stage fuses with a rapid explosion, the value of the criterion (16) should be maximized. Criterion (16) is of highly non-linear nature (in the power of 3), which guarantees a significant increase in the steepness of the current density rise in the forming stage fusible elements. Therefore, it is possible to integrate TSFF with energy sources and PFS with relatively low current dynamics (e.g., FCG or supercapacitor bank) without significant deterioration of the pulse forming process. Rapid disintegration of the forming stage (related to significant steepness of the current density rise) can be obtained by appropriate selection of equivalent cross-sections of the forming stage fusible elements in relation to the preparatory ones.

The ratio *n*_1_*S*_1_*/n*_2_*S*_2_ of the effective cross-sections of fusible elements also affects the deionization time of the plasma channel in the preparatory stage during the zero-current state after current commutation to the forming stage. This deionization time, equal to the average time *t*_*2AV*_ of current flow through the elements of the forming stage, can be determined with some approximation from Eq. (17).

17 $${t}_{2AV}\approx \frac{{I}_{max}}{3{\gamma }_{i}\kappa } \cdot {\left(\frac{{n}_{2}{S}_{2}}{{n}_{1}{S}_{1}}\right)}^{2},$$

where κ = *U*_*C*0_*/L* approximate average current slope in the PFS circuit depending on the initial capacitance voltage *U*_*C0*_ and the forming inductance *L* under the conditions of TSFF supply from the pulsed capacitor bank. In the case of TSFF cooperation with the FCG, the steepness of the current rise depends mainly on the FCG design.

In the case of high-density plasma formed after the disintegration of fusible elements, the deionization occurs as a result of recombination of charge carriers, i.e., electrons and ions. The deionization of the plasma channel depends largely on cooling conditions and the possibility of plasma channel expansion. Due to the deionization times considered at the level of hundreds of ns, the only effective mechanism for cooling the plasma channel formed after the disintegration of fusible elements is radiation, which is limited due to high-temperature conditions in the fuse chamber. Therefore, the dominant factor causing a rapid deionization and the increase of equivalent resistance of the plasma channel is the plasma radial expansion^45–48^, developing with a specific speed resulting from the dynamics of the fuse electro-explosion. This phenomenon causes a significant reduction in the density of free charge carriers in the plasma channel as a function of time. Thus, the higher the disintegration dynamics of fusible elements, the higher the subsequent deionization speed of the plasma channel.

The preliminary laboratory tests have indicated that, along with the obvious impact on the dynamics of the disintegration itself, the wire diameter of the forming stage fusible elements may also have a direct impact on the dynamics of radial expansion of the plasma channel in the time after the initiation of the disintegration process. For elements with smaller diameter, the speed of recovery of the electric insulation strength of the channel is greater than for elements with larger diameter (while maintaining the same equivalent cross-sections, and meeting the criterion of homogeneous heating resulting from the skin effect). The research into a qualitative model of this phenomenon is ongoing.

Premature disintegration of the forming stage fusible elements in relation to the beginning of current commutation decreases the zero-current time interval necessary to restore the electric strength of the preparatory stage plasma channel. In this case, when the possibility of adequate increase of the equivalent resistance *R*_*S1*_ is not ensured, the appearance of significant overvoltage after the electro-explosion of forming stage elements makes that the return current flow may take place again in the branch of the preparatory stage. The amplitude of this current is related to the duration of the zero-current time interval *t*_*2AV*_. The flow of the return current causes the internal energy of the preparatory stage plasma channel to increase again. If the critical value of the internal energy related to the thermal ionization energy of the channel is exceeded, the arc may re-ignite and the follow current may flow.

In a single-stage FF, in which the zero-current interval does not occur, the plasma channel resulting from low-dynamics disintegration of fusible elements has a limited possibility of restoring the electric recovery strength due to the continuous supply of thermal energy from the limited current. Consequently, as compared to TSFF, there is a much higher probability of arc reigniting after limiting the current to zero (or during the limitation process, on the falling slope of the current).

A summary of the main features and properties of TSFF compared to single-stage FF is presented in Table 1.

**Table 1:** Comparing features of two-stage and single-stage forming fuses.

Single-stage forming fuse	Two-stage forming fuse
The cross-section and number of parallel fusible elements results from a compromise between the value of the pre-arcing Joule integral (ensuring the maximum value of the forming coil current) and the rate of the fuse equivalent resistance increase during the disintegration of fusible elements	The current increase in the PFS forming coil is provided by the preparatory stage fusible elements, and the value of the commutated current (within a certain range) does not depend on the resistance of the forming stage elements High current limitation dynamics is ensured by rapid electro-explosion of the forming stage fusible elements Significant disintegration dynamics of the forming stage fusible elements is related to the high rate of the current density increase *dj/dt* after the commutation
The length of the single-stage FF fusible elements must be a compromise between the initial fuse resistance (limiting the increase steepness and the maximum value of the formed current) and the maximum generated overvoltage (related to the length of the plasma channel after the disintegration of elements)	It is possible to significantly extend the length and reduce the number and cross-section of the forming stage fusible elements in relation to the preparatory stage, which ensures the generation of higher overvoltages
The dynamics of electric recovery of the FF plasma channel is limited, hence there is a high probability of arc reignition, followed by the current flow discharging the energy remaining in the capacitor bank	After the current commutation to the forming stage, a free electric strength recovery of the preparatory stage plasma channel in the zero-current state occurs A very high speed of the forming stage electric strength recovery is related to the extremely high dynamics of the electro-explosion of fusible elements and significant decrease of the plasma channel density in a short time The risk of arc reignition after the forming process and the resulting current flow through the TSFF is much lower than in a single-stage FF
Integration with low-dynamics current sources causes a reduction in the pulse forming efficiency due to much lower steepness of the current density rise *dj/dt*	It can be integrated with low-dynamics current sources without significantly affecting the high-power pulse forming process

Summarizing, it can be stated that, due to its physical characteristics, the two-stage forming fuse with spark gap commutation provides much better high-power pulse forming operating parameters in a wide range of current–voltage conditions, compared to single-stage FF solutions.

## Laboratory tests of the two-stage forming fuse model

In order to verify the TSFF concept, a laboratory model has been developed and manufactured, as well as a number of laboratory tests have been performed in the proposed PFS supplied from a pulse capacitor bank. The aim of the experimental research was to confirm the properties of the TSFF determined on the basis of theoretical analysis, as well as to correct some design solutions.

### Description of the TSFF laboratory model

**Figure 11:**
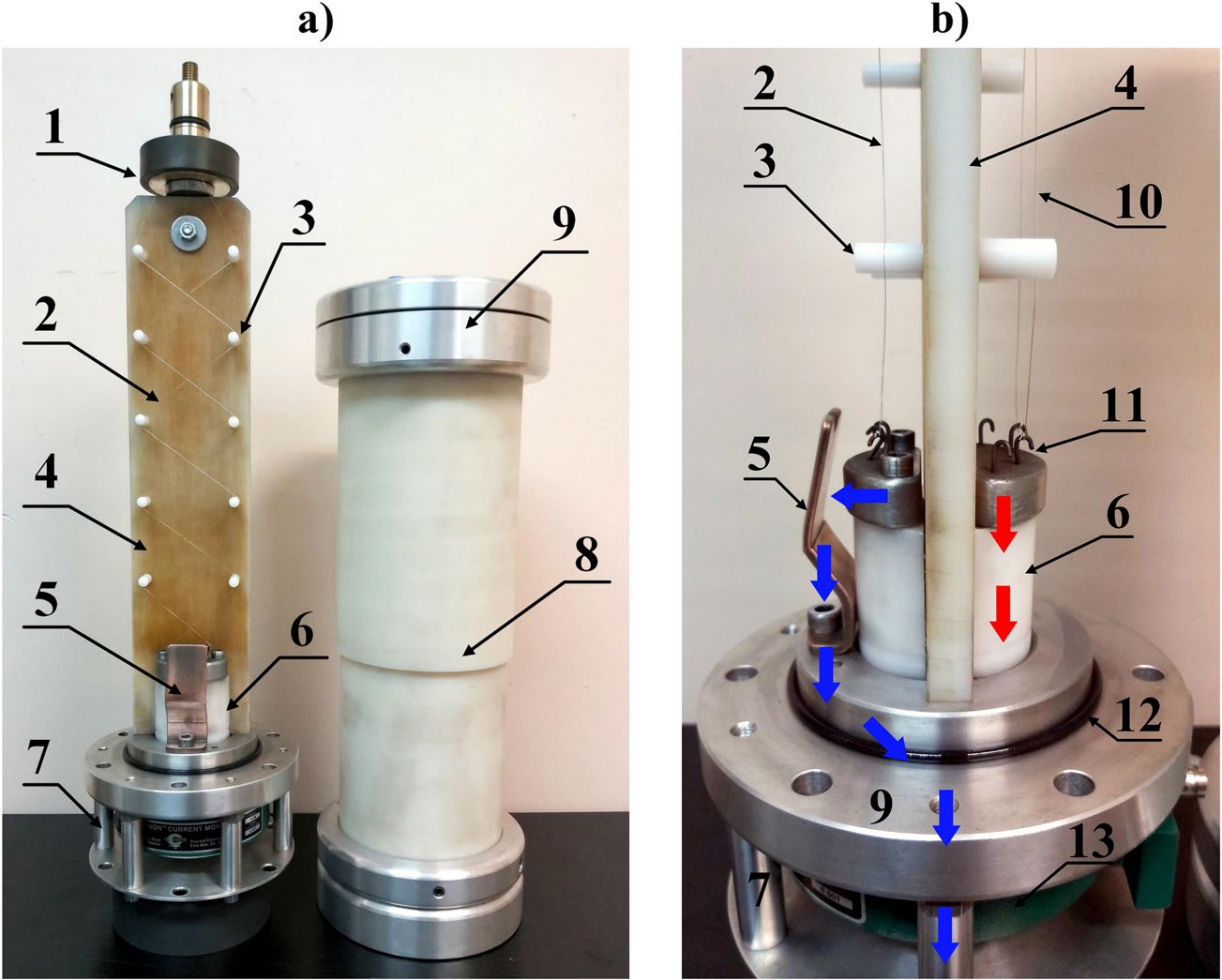
Photographs of the TSFF laboratory model: view from the side of the forming stage and commutation spark gap (**a**) and a profile with a close-up view of the commutation spark gap structure (**b**): 1—quick mounting system for fusible elements, 2—forming stage fusible elements, 3—insulating supports, 4—fuse compartment insulating partition, 5—CSG adjustable electrode, 6—bushing insulator and support for lower internal fitting of fusible elements, 7—external fuse cage with an integrated current measuring transducer for measuring fuse current components, 8—insulating tube of the fuse compartment, 9—external fuse fitting, 10—preparatory stage fusible elements, 11—lower internal fittings with hooks, 12—gas seal of the fuse compartment, 13—broadband pulse current measuring transducer. The approximate current flow path of the preparatory stage is marked with red arrows, while the blue arrows indicate the forming stage current flow path.

The photographs of the TSFF model showing its structure and characteristic elements are shown in Fig. 11.

The supporting structure for the fusible elements has been formed in a flat configuration, i.e., both stages of the FF are separated by an insulating barrier having the form of a plate made of polyoxymethylene material. The preparatory stage elements (with length of 320 mm) are suspended on one side of the barrier (as shown in Fig. 11), between the internal electrodes of the FF chamber. The insulating barrier plate supports the forming stage elements on the insulating rods, which allows mounting a polygonal-type winding of silver wires. The supporting structure with fusible elements is placed in the fuse compartment in the form of a polyamide tube with two external fittings attached to its ends. The use of insulating supporting rods and polygonal-type winding of the forming stage fusible elements allows to increase their length to *l*_2_ = 430 mm. When using a helical or polygonal winding method, the fusible elements of the same stage (preparatory or forming) are positioned in such a way as to ensure a uniform potential gradient (during the disintegration process) along their length, which means that any two close to each other points along the length of the fusible wires have a similar potential. This procedure is used to limit the probability of discharges ignition between the fusible elements. On the other hand, due to the possibility of disturbances in the structure of the fusible wire (which was additionally calibrated at the production stage), slight potential differences may occur. Therefore, the fusible elements should be separated from each other. The study successfully assumed a minimum distance of 5 mm. With the proposed method of fusible element winding (shown in Fig. 11a), the maximum number of elements of the preparatory stage of this particular TSFF laboratory model is equal to *n*_*1max*_ = 10, while for the forming stage *n*_*2max*_ = 6. Figure 11b presents a simplified design of the TSFF commutation spark gap having the form of a suitably shaped copper cathode and aluminum anode. Appropriate mounting of the copper electrode has made it possible to adjust the distance between the spark gap electrodes and, as a result, to adjust the ignition voltage *U*_*SGI*_ in the range from 50 kV to approx. 180 kV. The minimum value of the ignition voltage *U*_*SGI*_ must certainly not be lower than the supply voltage of the PFS circuit (as in the case of the presented paper—10 kV). If the *U*_*SGI*_ is not reached by the preparatory stage, no commutation will occur and TSFF will behave like a single-stage FF with the parameters of the preparatory stage. In the case when the CSG ignition takes place too early, that is before the fusible elements of the preparatory stage are disintegrated, the fusible elements of both stages will disintegrate roughly parallel in time and the achieved parameters will also be comparable to the single-stage FF.

In order to measure separately relevant components of the TSFF current (preparatory stage current *i*_*1*_ and forming stage current *i*_*2*_), an appropriate concentric current divider has been installed in the lower part of the model. The internal path of this divider conducts only the preparatory stage current component (through the use of an insulation bushing inside the fuse chamber), while the outer path (current cage) conducts only the current of the forming stage. Figure 11b shows the flow paths of the respective fuse current components. In the case of unavailability of a current transducer with appropriate measurement range, the proposed geometric solution makes it possible to extend the current measurement range by using the topology of the current divider together with a suitable measurement compensation method^49^.

Apart from the parameters of the PFS circuit in which the FF is installed, the efficiency of fuse-based high-power pulse forming is influenced by many design factors of the fuse, including:

- cross-section and geometry of a single fusible element, and the number of parallel elements,
- length of the fusible elements,
- material properties of the fusible elements,
- type and pressure of the medium (insulating gas) filling the fuse compartment.

Optimal selection of the above FF parameters to ensure proper effectiveness of the pulse forming process is a problem of nonlinear multi-criteria analysis and is not the aim of this paper. Work is underway on a detailed specification of the impact of selected TSFF operating parameters on the pulse forming process.

### Test conditions and methods

The laboratory tests of the TSFF have been carried out in a laboratory PFS supplied by the pulse capacitor bank. The forming system was tested in the no-load condition so as not to introduce additional parameters that could affect the operation of the TSFF and for easier comparison with other PFSs described in the literature.

**Figure 12:**
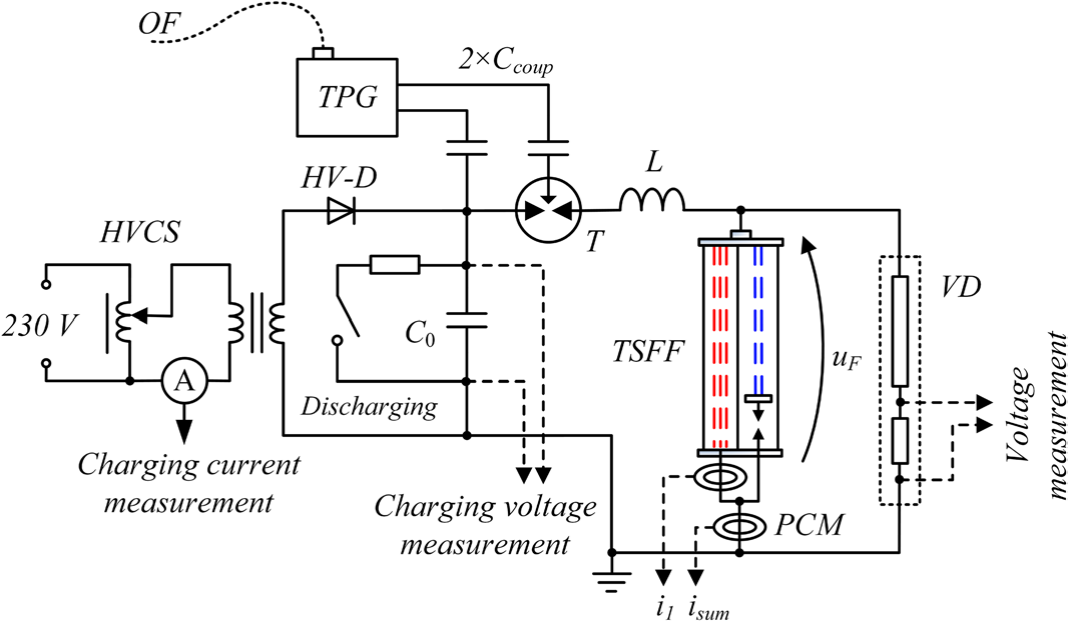
Schematic diagram of the TSFF-based PFS laboratory test stand: *C*_0_ = 200 μF—battery of pulse capacitors charged to initial voltage *U*_*C0*_ = 10 kV, *T* trigatron, *L* = 2.8 μH—forming inductance, *VD* pulse voltage divider, *PCM* Pearson pulse current monitor, *HV-D* high voltage diode stack; *HVCS* high voltage charging system, *TPG* triggering pulse generator, *OF* optic fiber, *C*_*coup*_ high voltage coupling capacitors, *i*_*1*_, *i*_*sum*_ current of the preparatory stage and total current of the fuse, *u*_*F*_ voltage across the fuse.

**Figure 13:**
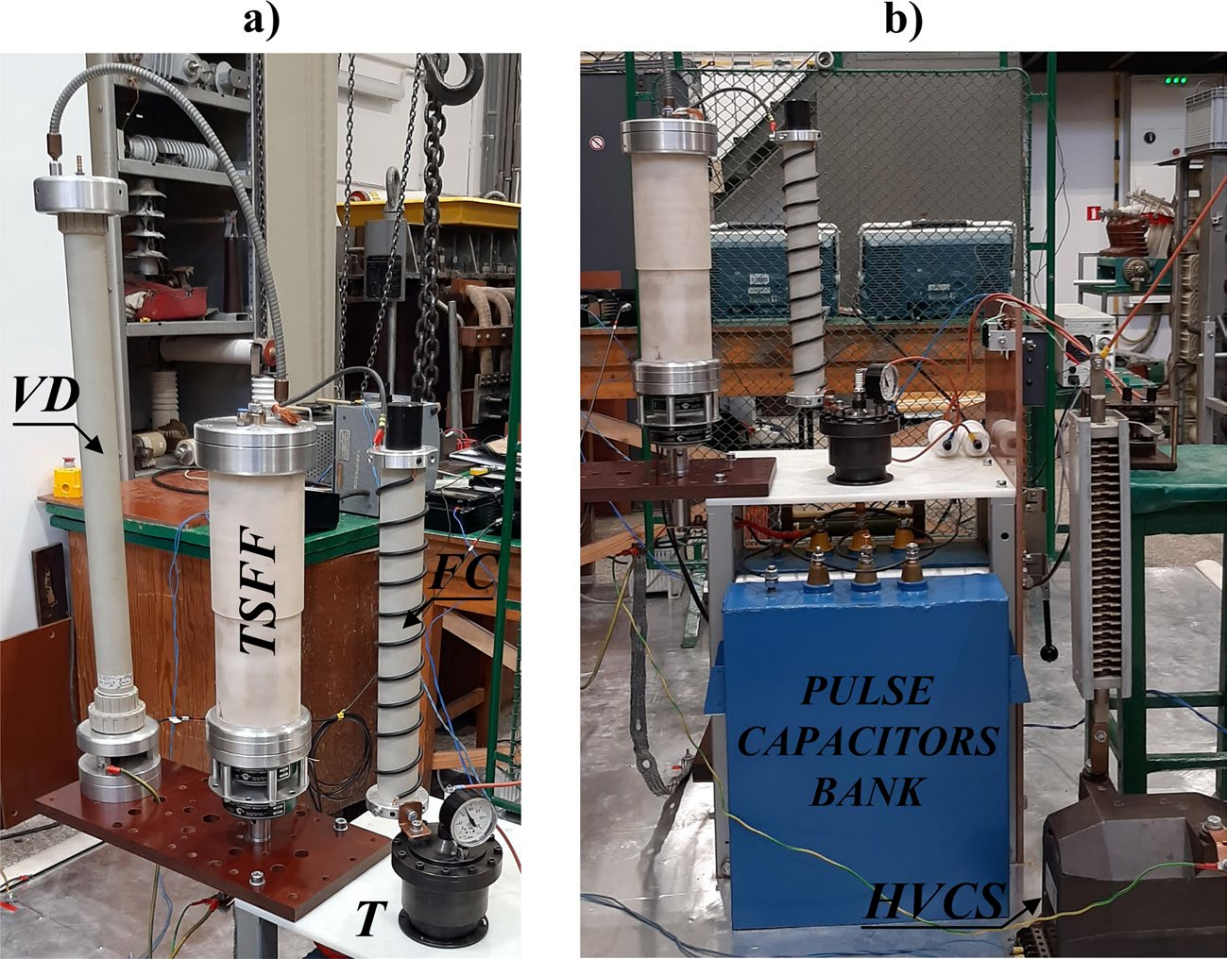
Photographs of the TSFF-based PFS laboratory test stand supplied from a battery of pulse capacitors. Designations according to Fig. 12.

The laboratory test stand includes an energy source in the form of a capacitor bank with capacity of *C*_0_ = 200 µF and low internal parasitic inductance (less than 100 nH—the value estimated by measuring the short-circuit current waveform), pre-charged to *U*_*C0*_ = 10 kV. Other elements of the PFS include a trigatron *T* (electrically triggered spark gap), coreless forming coil (FC) with inductance *L* = 2.2 µH, and the TSFF model. The schematic diagram of the test stand is presented in Fig. 12, and the photographs of the stand—in Fig. 13.

The TSFF voltage was measured using a resistive pulse voltage divider VD with the frequency bandwidth from DC to approx. 10 MHz manufactured, tested and verified at the Gdansk University of Technology. Measurement of the currents flowing in individual TSFF stages was performed with the use of broadband Pearson current transducers^50^ with appropriate ranges and measurement bands, i.e. Pearson Current Monitors model 5624 (20 MHz, 20 kA) and model 4191 (7 MHz, 50 kA). To enable the measurement of current components in both TSFF stages, an insulating bushing which conducted the preparatory stage current of the TSFF through the lower fuse fitting was installed at the bottom part of the fuse chamber and connected to the external cage-type current path outside the fuse compartment. This way, both the total TSFF current and the current of the preparatory stage could be measured during the tests. The forming stage current waveforms were determined numerically as the difference between the two measured components. The signals from all measuring transducers, i.e., current transducers and voltage dividers, were recorded with separate and galvanically insulated oscilloscopes (Tektronix MSO58, DPO4104 and DPO 4054 class or higher) due to the occurrence of potential differences between the parts of the circuit in which they were installed and to minimize crosstalk between the oscilloscope channels.

### Laboratory test results

**Figure 14:**
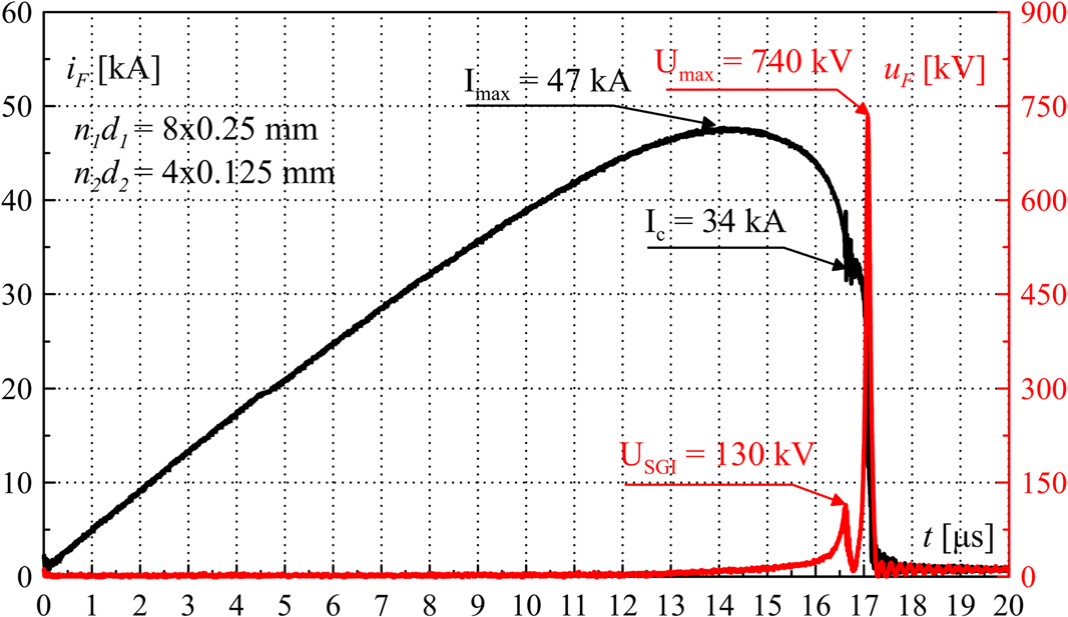
TSFF current *i*_*F*_ and voltage *u*_*F*_ waveforms during operation—laboratory test results. Characteristic values are marked in the figure.

**Figure 15:**
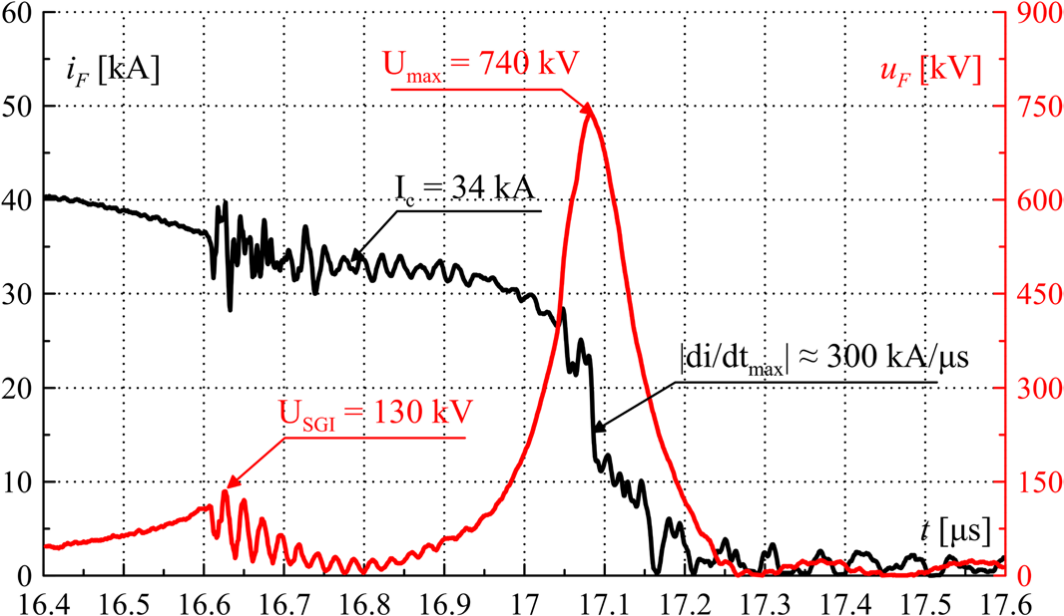
TSFF current *i*_*F*_ and voltage *u*_*F*_ waveforms during operation—laboratory test results. Characteristic values are marked in the figure. Close-up on the commutation and the current limiting process.

**Figure 16:**
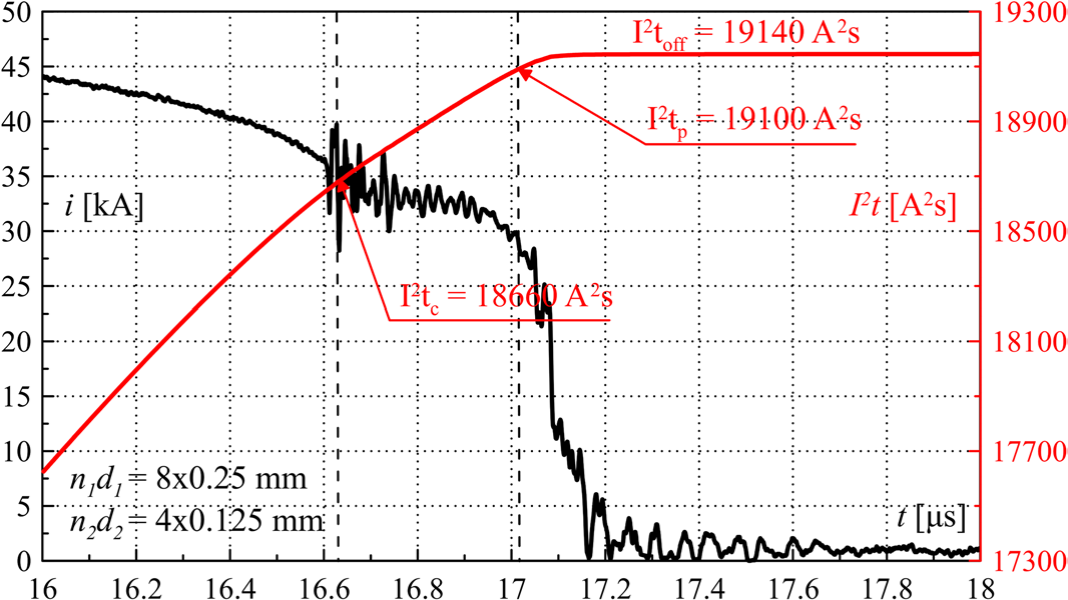
TSFF current *i*_*F*_ and Joule integral *I*^2^*t* waveforms during operation—laboratory test results. Characteristic values are marked in the figure. Close-up on the commutation and the current limiting process.

**Figure 17:**
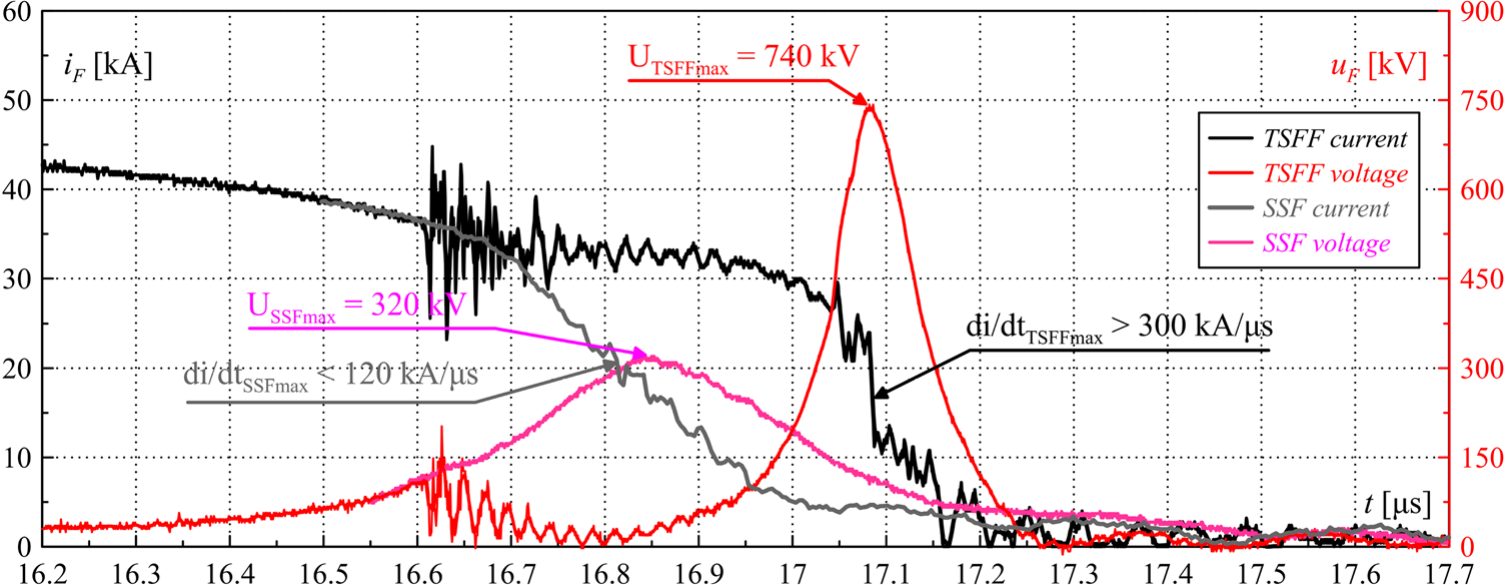
Comparing waveforms of the TSFF and single-stage fuse (SSF) current *i*_*F*_ and voltage *u*_*F*_ during operation—laboratory test results. Characteristic values are marked in the figure. Close-up on the current limiting process.

Figures 14, 15 and 16 present the waveforms of current *i*_*F*_*(t)*, voltage *u*_*F*_*(t)*, and Joule’s integrals *I*^2^*t* computed as in (6). They were determined on the basis of experimental tests of the TSFF laboratory model with the following parameters: *n*_1_ = 8, *n*_2_ = 4, *U*_*SGI*_ = 130 kV. For these parameters, the maximum value of the generated overvoltage reached approx. 740 kV, which is more than twice as high as in the case of PFS using a single-stage fuse under similar current conditions and similar parameters of fusible elements (Fig. 17).

Figure 15 shows the waveforms of fuse current and voltage, with a close-up view on the commutation and current limitation process leading to significant and rapid overvoltage formation. The maximum absolute value of the current limitation dynamics *di/dt*_*max*_ was approx. 300 kA/μs. Figure 16 shows the waveform of the TSFF Joule integral, on the basis of which it is possible to determine the approximate values of the specific pre-arcing and switching-off integrals of the forming stage fusible elements as the difference of the pre-arcing integral *I*^2^*t*_*p*_ of the entire TSFF and the integral at the time of interstage current commutation *I*^2^*t*_*c*_ (similarly, *I*^2^*t*_*off*_ in the case of the switch-off integral). In the presented variant, the values of the pre-arcing integral and the switching-off integral of the forming stage related to the square of the cross-section area of the elements was calculated as:

18 $${h}_{p2} =\frac{{I}^{2}{t}_{p}-{I}^{2}{t}_{c}}{{\left({n}_{2}{S}_{2}\right)}^{2}} \approx 1.9\times {10}^{17}\frac{{A}^{2}s}{{m}^{4}},$$

19 $${h}_{off2}=\frac{{I}^{2}{t}_{off}-{I}^{2}{t}_{c}}{{\left({n}_{2}{S}_{2}\right)}^{2}} \approx 1.99\times {10}^{17}\frac{{A}^{2}s}{{m}^{4}}.$$

The value of the proper switching-off integral is greater by approx. 5% than that of the pre-arcing integral. A very small difference in the presented values proves a very high dynamics of disintegration of the forming stage fusible elements. These values can be compared to the Meyer constant, as they have the same physical meaning, confirming almost double increase in the heat accumulated in the fusible elements of the forming stage during such a violent electro explosion, in comparison with typical values available in the literature (concerning, for example, TSFF preparatory stage or single-stage FF fusible elements)^29,38^ as presented below Eq. (6).

Figure 17 compares the results of the pulse forming process and overvoltage generation in the form of fuse current *i*_*F*_ and voltage *u*_*F*_ waveforms for two FF technologies: single-stage fuse and TSFF with the same number of fusible elements: *n*_*SSF*_ for a single-stage fuse, and *n*_*TSFF1*_ for the preparatory stage (in the case of TSFF), *n*_*SSF*_ = *n*_*TSFF1*_ = 8.

The laboratory results have fully confirmed the effectiveness of the proposed concept. During the operation of both FFs with the same parameters of the PFS, more than 2.3-fold amplification of the generated overvoltage, with corresponding reduction of pulse duration, was achieved in the case of using TSFF, as compared to a single-stage FF. The use of TSFF has made it possible to achieve an overvoltage pulse steepness in the order of 10,000 kV/μs.

**Figure 18:**
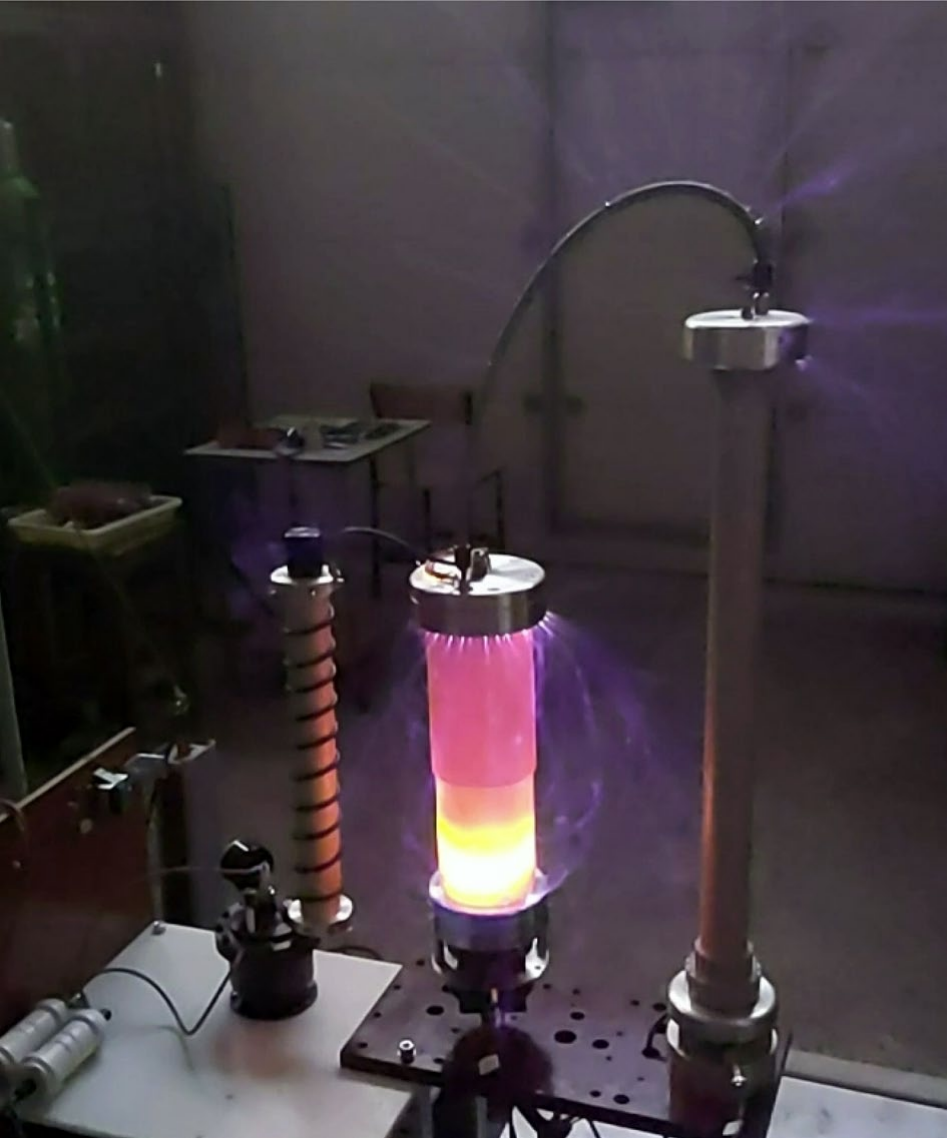
View of partial discharges of volatile nature in the space around the TSFF model, voltage divider, and the upper fitting of the forming coil during the pulse forming process (for the overvoltage peak value *U*_*max*_ = 740 kV).

In the course of the TSFF-based PFS laboratory research, the tests were recorded using a high-speed camera, which allowed studying the phenomenon of partial discharges (air partial ionization) in the space around the fuse, voltage divider, and the upper fitting of the forming coil during the pulse forming process (for the overvoltage peak value *U*_*max*_ = 740 kV). A selected frame from this recording is shown in Fig. 18. Due to high electric field intensity in the space around the TSFF model, it was necessary to use high-purity insulating materials with very high electrical strength.

The tests carried out with the laboratory model of a two-stage forming fuse have proven that its design, combined with appropriate selection of parameters (equivalent cross-sections of the preparatory and forming stage fusible elements and the voltage of the commutation spark gap), allows for greater steepness of current limitation to zero in PFS and greater electric return strength of the fuse compared to the previously used single-stage FFs. Consequently, the TSFF allows the formation of an overvoltage pulse with a much greater steepness and maximum value.

## Conclusions and discussion

The proposed concept and construction of the TSFF enables the achievement of high-power pulse forming parameters, much more favorable than those offered by conventional single-stage FFs. The physical principle of operation and the parameters of TSFF have been confirmed in tests carried out in laboratory conditions on the designed and manufactured prototype, during which an overvoltage of approx. 740 kV was achieved for the fuse column length of 350 mm, which is more than twice as high as in the case of using single-stage FFs of any configuration.

Based on the results of experimental tests (current and voltage waveforms), it is possible to determine the maximum instantaneous power of the generated pulse and the power density of the system as the universal indicator that determines the effectiveness of the high-power pulse forming process. In the above tests, the peak power reached the value of *p*_*max*_ ≈ 14,800 MW, while the power density (taking into account all elements of the laboratory stand) was approx. *p*_*max*_*/V* ≈ 61 GW/m^3^. These values are significantly greater than in the case of using, for example, a Marx generator, even with additional forming systems (*p*_*max*_ < 6000 MW), or PFS with conventional single-stage fuses (*p*_*max*_ < 5000 MW for similar supply parameters).

Table 2 presents the parameters obtainable in various technologies of compact forming systems which have been collected on the basis of the analysis of available literature sources. Due to the comparison of the parameters of the generation and forming systems of different technologies, this comparison is illustrative in nature.

**Table 2:** Maximum achievable parameters of high power pulse generation and forming systems—comparison of the compact technologies and state of the art.

Pulse generation technology	Pulse forming technology	Max. overvoltage^1^ [kV]	Generated pulse power^1^ [MW]	Pulse equivalent energy [kJ]	Estimated equivalent power density [GW/m^3^]	Ref.
Marx generator	–	200 ÷ 500	< 3000	0.01 ÷ 1	< 10	^15,17^
Marx generator	Blumlein line	400 ÷ 600	< 6000	0.1 ÷ 1	< 25	^11,51^
FCG	–	< < 100	1000 ÷ 10,000	5 ÷ 30	< 600	^25,30,52^
FCG	Electro-explosive FF	200 ÷ 400	5000 ÷ 30,000	5 ÷ 30	< 1200	^27,30–32^
Pulse capacitors	Electro-explosive FF	150 ÷ 400	< 5000	0.1 ÷ 10	< 20	^30,33,53,54^
Pulse capacitors	TSFF	700 ÷ 800 and more	~ 15,000	1 ÷ 10	> 60	This work

^1^Defined as the absolute maximum values of the generated pulse at the optimal load (Marx generators) or without load (in the case of FF technology).

It should also be noted that when using TSFF, it is possible to scale the system by increasing the number of parallel fusible elements (proportionally for the preparatory stage and the forming stage) and increasing the input current from the previous generation stage. Unlike other technologies (including single-stage FF), TSFF provides a possibility of using a wide range of current sources without losing the ability to form significant overvoltages, as it can operate with current sources with a significantly reduced current rise steepness, such as supercapacitor banks or FCGs.

Advanced work on the development of TSFF technology is currently underway, including numerous laboratory tests and simulation studies. TSFF has a great development potential, mainly towards applications and integration with current sources with high energy density (e.g., FCG for current and energy amplification phenomenon). Obtaining such significant overvoltages with a very small volume, and thus a significant power and energy density, creates new application possibilities, mainly in the field of directed energy, counter-drone systems, electromagnetic compatibility testing, or laboratory research requiring sources of high-power pulses.

At the same time, the obtained dynamics of disintegration of fusible elements, previously unattainable, may enable better understanding of the processes taking place during rapid phase changes of metals.

## Data Availability

The datasets used and/or analyzed in the current study are available from the corresponding author on reasonable request.
